# Augmented reality-based navigation system applied to tibial bone resection in total knee arthroplasty

**DOI:** 10.1186/s40634-019-0212-6

**Published:** 2019-11-11

**Authors:** Sachiyuki Tsukada, Hiroyuki Ogawa, Masahiro Nishino, Kenji Kurosaka, Naoyuki Hirasawa

**Affiliations:** Department of Orthopaedic Surgery, Hokusuikai Kinen Hospital, 3-2-1 Higashihara, Mito, Ibaraki, 310-0035 Japan

**Keywords:** Knee, Arthroplasty, Computer assisted surgery, Navigation, Smartphone, Virtual reality

## Abstract

**Background:**

This pilot study was performed to examine the accuracy of the AR-KNEE system, an imageless navigation system using augmented reality (AR) technology for total knee arthroplasty. The AR-KNEE system enables the surgeon to view information from the navigation superimposed on the surgical field on a smartphone screen in real time.

**Methods:**

Using the AR-KNEE system, one surgeon resected 10 tibial sawbones with viewing the tibial axis and aiming varus/valgus, posterior slope, internal/external rotation angles, and resection level superimposed on the surgical field. We performed computed tomography of the resected sawbones and measured the varus/valgus, posterior slope, and internal/external rotation angles using a designated computer software. The thickness of the resected bone was measured using digital calipers.

**Results:**

The absolute differences between the values displayed on the smartphone screen and the measurement values for varus/valgus, posterior slope, internal/external rotation angles, and thickness of the resected bone were 0.5° ± 0.2°, 0.8° ± 0.9°, 1.8° ± 1.5°, and 0.6 mm ± 0.7 mm, respectively.

**Conclusions:**

This pilot study using sawbones suggested that the AR-KNEE system may provide reliable accuracy for coronal, sagittal, and rotational alignment in tibial bone resection during total knee arthroplasty.

## Background

Although computer navigation for total knee arthroplasty (TKA) can improve limb alignment and may reduce the revision rate (de Steiger et al. 2015), its lack of cost-effectiveness remains a challenge to its widespread adoption (Novak et al. 2007; Slover et al. 2008). The need for the surgeon to take their eyes off the surgical field to view the monitor is another issue that must be addressed in the further development of computer navigation.

In total hip arthroplasty (THA), Ogawa et al. developed a novel navigation system using augmented reality (AR) technology, in which the surgeon uses a free application installed on a smartphone (Ogawa et al. 2018). AR-based navigation allows the surgeon to view the information from the navigation superimposed on the surgical field through the smartphone. Putting the smartphone in a sterilized case enables the surgeon to use AR-based navigation without taking their eyes off the surgical field in a sterile setting. There have been no previous reports regarding the use of an AR-based navigation system for TKA.

For TKA, we developed an AR-based navigation system (AR-KNEE system) that was applied to tibial bone resection. As the surgeon uses an application installed on their own smartphone, no expensive equipment is required when using the AR-KNEE system. The surgeon can easily determine the target angles of varus/valgus and posterior slope by pushing the icons on the smartphone display. The AR technology allows the surgeon to view these angles as reference lines superimposed on the real surgical field on the smartphone display.

In this pilot study, we introduce the AR-KNEE system for tibial bone resection. The aim of this preclinical study was to assess the accuracy of the AR-KNEE system in tibial bone resection using sawbones. We hypothesized that the AR-KNEE system would provide reliable accuracy for coronal, sagittal, and rotational alignment in tibial bone resection during TKA.

## Methods

### AR-KNEE system

**Fig. 1:**
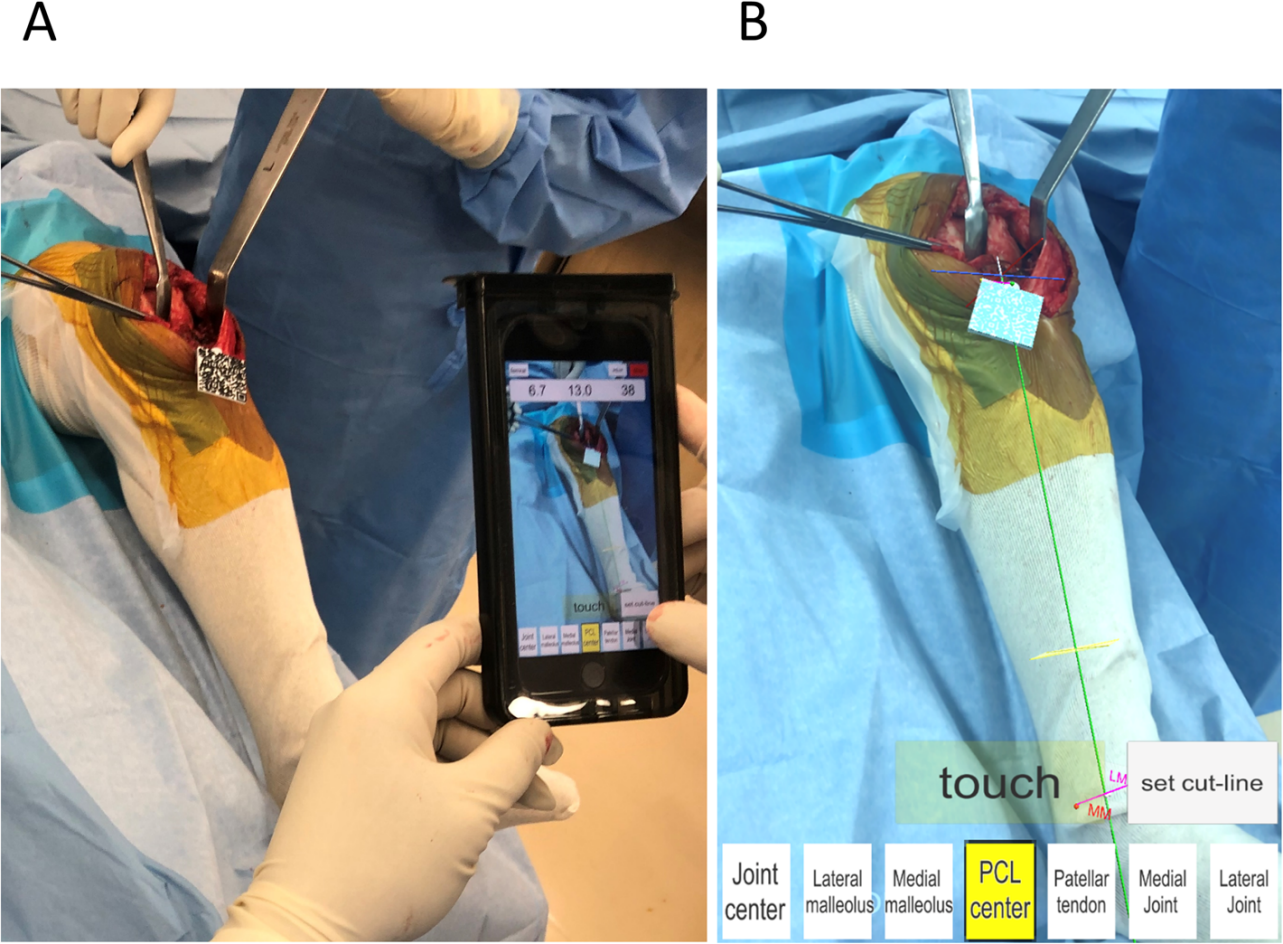
Total knee arthroplasty using the AR-KNEE system. **a** The surgeon viewed the information superimposed on the surgical field through the screen of the smartphone in real time. Guide marker (resin marker with quadrate two-dimensional bar code) was fixed to the proximal tibia. The smartphone was placed in a sterilized case. **b** Screenshot image of (**a**). The tibial axis was superimposed on the patient’s lower leg (green line). When the smartphone camera recognized the two-dimensional bar code, the color of the marker guide turned light blue on the screen

AR technology projects computer-generated images onto the real world (Ogawa et al. 2018). The AR-KNEE system allows the surgeon to view the tibial axis superimposed on the surgical field through the display of the smartphone. The AR-KNEE system also enables the surgeon to view the aiming varus/valgus angle and posterior slope angle superimposed on the surgical field (Fig. 1). Similar to conventional computer navigation, the AR-KNEE system provides real-time information during surgery and intraoperative feedback.

**Fig. 2:**
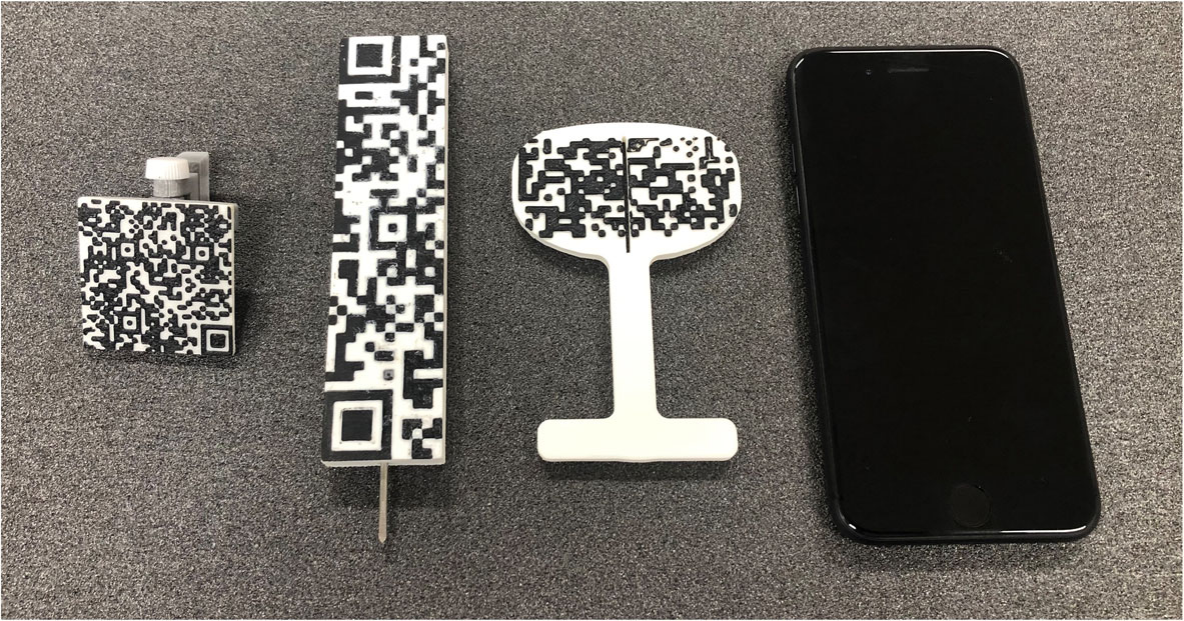
Markers and smartphone used in the AR-KNEE system. From left to right: guide marker, pointer, oval marker, and smartphone. The camera of the smartphone recognizes two-dimensional bar codes of guide marker, pointer and oval marker when using the AR-KNEE system. Note that the oval marker has a slit that allows the surgeon to mark the tibial rotational alignment reference line

The AR-KNEE system consists of a guide marker, pointer, oval marker, and the surgeon’s own smartphone (Fig. 2). The markers are made of acrylonitrile-butadiene-styrene resin and created using a three-dimensional printer for home use.

The guide marker is fixed to the proximal tibia through the same skin incision as the surgical approach (Fig. 1). The guide marker is designed to be placed proximal to the superior margin of pes anserinus. According to the surgeon’s preference, the guide marker can be fixed to the proximal tibia through separate small incision.

**Fig. 3:**
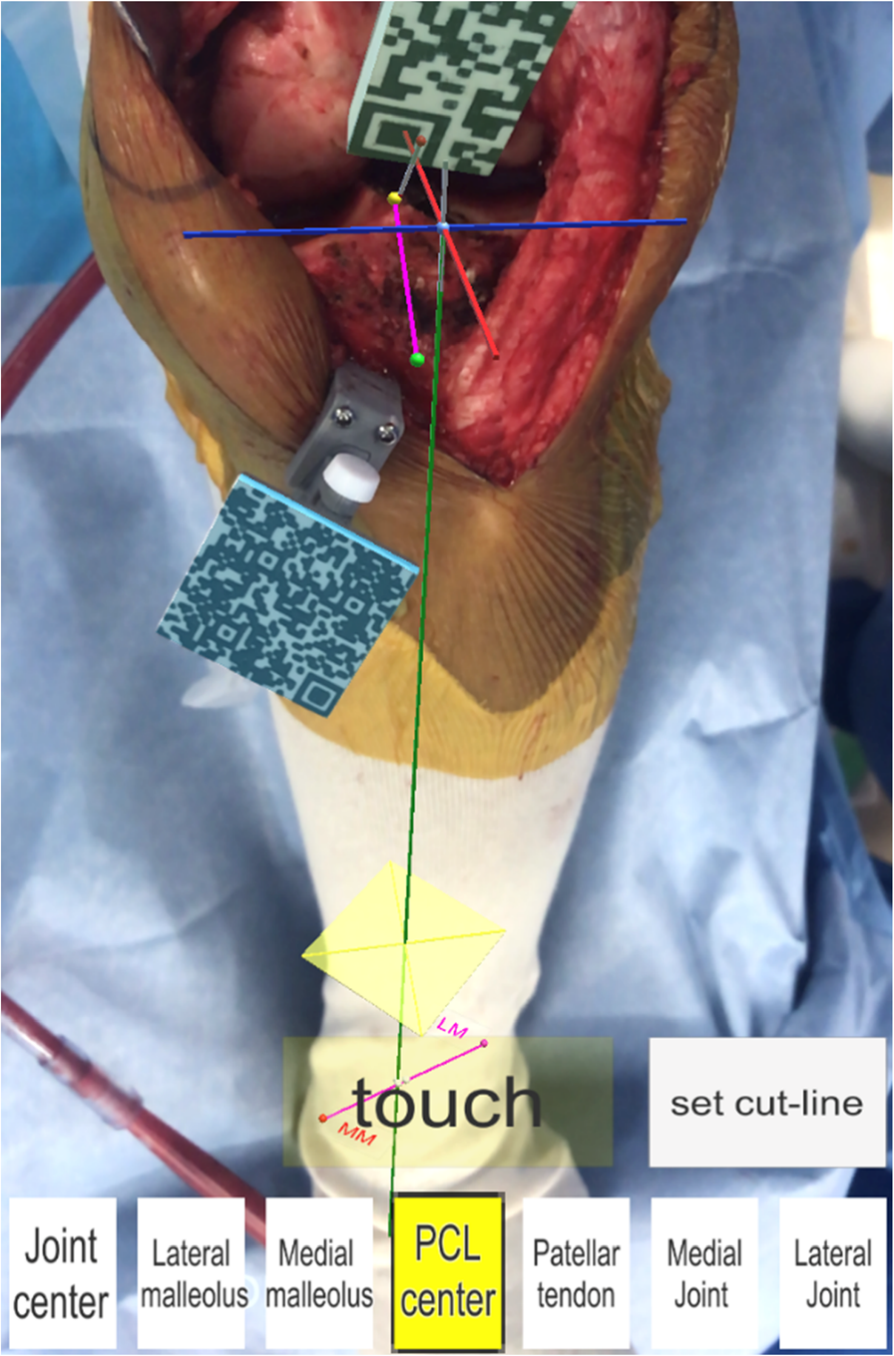
Registration of the bone landmarks in the AR-KNEE system. The surgeon registered bone landmarks using the pointer (the pin attached to the resin marker with rectangular two-dimensional bar code). The AR-KNEE system visualized the tibial long and the anteroposterior axes as green and pink lines, respectively

The smartphone camera recognizes the guide marker (Fig. 1). The surgeon registers bone landmarks using the pointer (Fig. 3). The smartphone mathematically interprets the positional relationships between the guide marker and bone landmarks, and creates a tibial coordinate system. The position of the smartphone in relation to the lower limb during registration is not associated with the accuracy of the acquired data. The smartphone displays the reference lines including tibial long and anteroposterior axes of the tibial coordinate system (Figs. 1, 3).

**Fig. 4:**
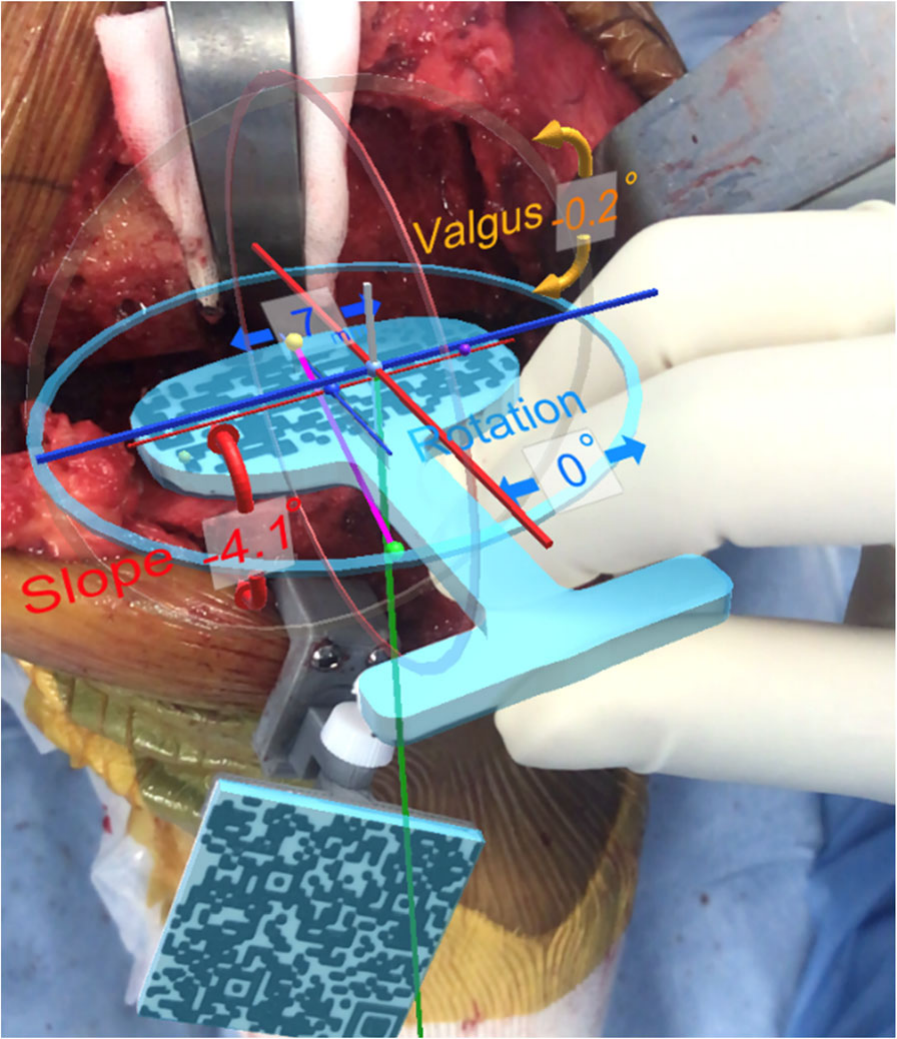
Confirmation of the varus/valgus, posterior slope, and rotation angles after bone resection using the AR-KNEE system. The surgeon checked the angles of tibial resection by placing the oval guide (resin marker with oval two-dimensional bar code) on the resected bone surface. In this case, the AR-KNEE system showed a varus angle of 0.2°, posterior slope of 4.1°, and external rotation of 0°

After bone resection, the surgeon can confirm the varus/valgus, posterior slope, and rotation angles using the oval marker (Fig. 4).

### Surgical technique of AR-KNEE system

AR-KNEE utilizes tibial long and anteroposterior axes to uniquely determine the positions of the points in the surgical field. Three lines constituting the 3-dimensional coordinate system of AR-KNEE are (1) the tibial long axis, (2) the tibial anteroposterior axis, and (3) the cross-product of these two tibial axes. These tibial axes are created by registration of bone landmarks. Using the pointer, the following bone landmarks are registered: (1) the most prominent point of the medial malleolus, (2) the most prominent point of the lateral malleolus, (3) the tibial center on the tibial plateau, (4) the mediolateral center of the tibial insertion of the posterior cruciate ligament, (5) the medial border of the patellar tendon attachment, (6) the medial compartment of the tibial plateau, and (7) the lateral compartment of the tibial plateau.

The tibial long axis is determined from both the ankle center and the tibial center on the tibial plateau. In the AR-KNEE system, the center of the ankle is determined from the medial and lateral malleolus. The definition of the ratio of the ankle center from the lateral malleolus to the medial malleolus can be changed to suit the surgeon’s preference. In this study, the ankle center was calculated by dividing the digitized transmalleolar axis according to the ratio of 55% lateral to 45% medial (Kim et al. 2005). The definition of the tibial center of the tibial plateau can be determined according to the surgeon’s preference. In this study, the tibial center on the tibial plateau was defined as the bisection of the transverse tibial axis in this study (Stiehl 2007).

The anteroposterior axis of the tibia was determined by a line connecting the middle of the posterior cruciate ligament to the medial border of the patellar tendon attachment (Akagi et al. 2004).

The registration of medial and lateral compartments of the tibial plateau is used for the resection level of the tibia allowing the surgeon to view the proximal tibia resection level. A single point on each plateau is used to determine the resection level. The AR-KNEE system calculates the length of the line perpendicular from the reference point to the resection plane.

After completing registration, the AR-KNEE system enables the surgeon to view the reference lines superimposed on the tibia on their smartphone display (Fig. 1). The surgeon can also view the angles of varus/valgus and posterior slope on the display. The surgeon fixes the tibial resection block while viewing these angles. The tibia is resected through the slit of the tibial resection block using a bone saw in the standard manner. Placing the oval marker on the cut bone surface, the surgeon can intraoperatively confirm the angles of varus/valgus, posterior slope, and rotation (Fig. 4). The oval marker has a slit in the central part, which the surgeon use to mark the reference line of tibial rotational alignment using the slit (Fig. 2).

### Accuracy verification of AR-KNEE system using sawbones

**Fig. 5:**
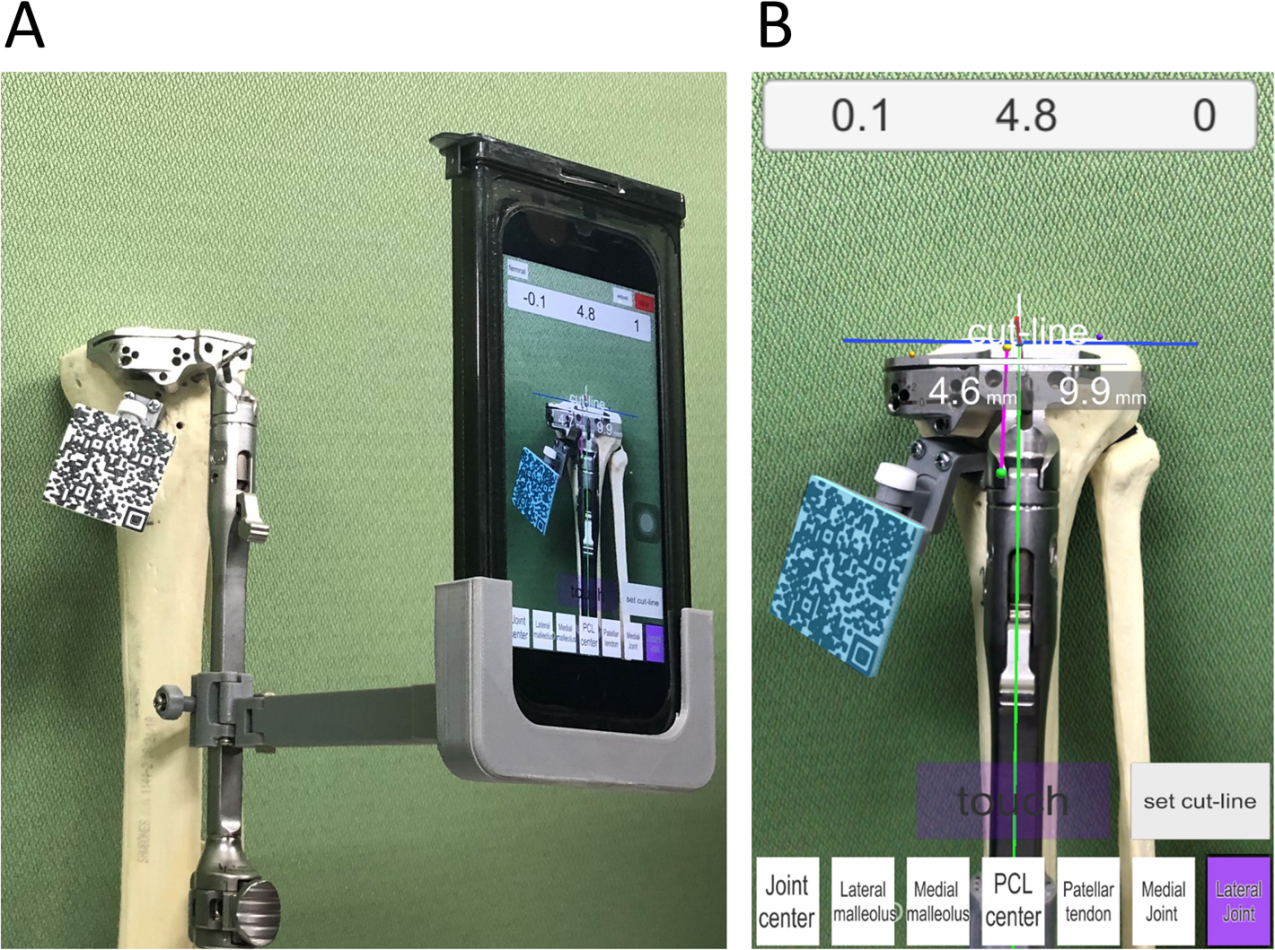
Resection of the proximal tibia of sawbone using the AR-KNEE system. **a** We cut the sawbone viewing the cutting angle and cutting amount of the bone following registration of the bone landmarks. The smartphone can be attached to the tibial bone resection guide as one option. **b** Screenshot image of (**a**). The AR-KNEE system showed the cutting block placed on the following parameters in this case: (1) varus angle of 0.1° on the long tibial axis (green line), (2) posterior slope of 4.8° on the long tibial axis (green line), (3) internal rotation angle of 0° on the anteroposterior axis (pink line), (4) 4.6-mm of bone resected from the medial tibia plateau, and (5) 9.9-mm of bone resected from the lateral tibia plateau. The white line indicates the resection level

This study was approved by the institutional ethics board. A total of 10 pairs of tibia and fibula sawbones were used to assess the accuracy of the AR-KNEE system (Fig. 5). A single knee surgeon (ST) resected the proximal tibia using the AR-KNEE system. The target values of tibial resection in this study were as follows: (1) perpendicular to the tibial long axis in the coronal plane; (2) 5° posterior slope in the sagittal plane; and (3) 10-mm resection from the lateral tibia plateau. Prior to registration of the bone landmarks, we made a small hole at each registration point using a 1.2-mm K-wire to minimize measurement error using computed tomography (CT). The small holes can be detected easily in CT images.

First, we measured the amount of bone resection using digital calipers (Monotaro, Hyogo, Japan) considering the thickness of the bone saw used in this study. The thickness of the bone saw was 1.27-mm. The resected proximal fragment including the tibia plateau was then attached to the main fragment of the tibia using 1.27-mm double-sided adhesive tape. The width of the adhesive tape was also the same as that of the bone saw. As the measurement of alignment using CT images is affected by the cutting plane, we referred to the standardized coordinate system using specifically designed measurement software (ZedKnee; LEXI, Tokyo, Japan) (Miura et al. 2018). We performed CT of the tibia and fibula pairs and entered the CT data in Digital Imaging and Communications in Medicine (DICOM) format into the ZedKnee software, which allows measurement of the angle of bone resection according to the coordinate axis defined by each investigator (Mochizuki et al. 2018). We defined the tibial long axis as a line connecting the tibial center on the tibia plateau and ankle center. The ankle center was defined as the point dividing the transmalleolar axis according to the ratio of 55% lateral to 45% medial (Kim et al. 2005). We defined the tibial anteroposterior axis as a line connecting the middle of the posterior cruciate ligament to the medial border of the patellar tendon attachment according to Akagi et al. (2004). Two investigators (HO and MN) independently measured varus/valgus angle in the coronal plane, posterior slope in the sagittal plane, and rotational alignment of the tibia.

We calculated the differences between the values displayed on the smartphone during use of the AR-KNEE system and measured values with CT or digital caliper.

### Statistical analyses

This was a pilot study to assess the accuracy of tibial bone resection using the AR-KNEE system. We calculated the absolute values of the differences between angles measured using CT and angles displayed on the smartphone screen in terms of varus/valgus, posterior slope, and rotation angles. In addition, we calculated the absolute values of differences between the thickness of the resected tibia measured using digital calipers and target thickness of 10.0 mm. The averages, standard deviations, and interquartile ranges were calculated for each parameter. We compared the varus/valgus, posterior slope, and rotation angles displayed on the smartphone screen and the measurement values determined by CT using the Mann-Whitney U-test.

To test interobserver reliability, the intraclass correlation coefficients of postoperative measurements of bone resection angles were calculated for two assessors (HO and MN).

## Results

Table 1 summarizes differences between the values displayed on the smartphone screen and the actual measurement values in terms of varus/valgus angle, posterior slope angle, internal/external rotation angle, and the thickness of the resected bone. There were no significant differences between the angles displayed on the smartphone screen and the measurement angles determined using CT (*P* = 0.27, 0.06, and 0.10 in varus/valgus, posterior slope, and rotation angle, respectively).

**Table 1:** Absolute values of differences between the values displayed on the smartphone screen and the actual measurement values

	Mean	Standard deviation	Interquartile range
Varus/valgus, °	0.5	0.2	0.4–0.6
Posterior slope, °	0.8	0.9	0.2–0.9
Internal/external rotation, °	1.8	1.5	0.9–2.2
Resected bone thickness, mm	0.6	0.7	0.2–0.8

The intraclass correlation coefficients between two assessors for varus/valgus, posterior slope, and rotation angles were 0.97 (95% CI, 0.92 to 0.99), 0.94 (95% CI, 0.81 to 0.98), and 0.79 (95% CI, 0.40 to 0.94), respectively.

## Discussion

The most important finding of this study was that the AR-KNEE system indicated varus/valgus and posterior slope angles of less than 1° and internal/external rotation angle < 2° for the differences between the values displayed on the smartphone screen and the actual measurement values with regard to bone resection of the proximal tibia using sawbones.

AR technology has been applied to various surgical procedures (Ogawa et al. 2018; Abe et al. 2013; Eftekhar 2016; Conrad et al. 2016). In TKA, one AR assisted system has been proposed in which the image of the bone exposed in the surgical field is intraoperatively projected to the image obtained by preoperative CT using an optical camera and computer (Pokhrel et al. 2019). Our AR-KNEE system is completely different from the system of Pokhel et al. (Pokhrel et al. 2019). The AR-KNEE system does not require preoperative CT and can superimpose tibial long and anteroposterior axes on the surgical field using smartphone and marker with two-dimensional bar codes. When the line does not fit the tibia properly, the surgeon can easily recognize that the registration is incorrect.

This pilot study was performed to assess the accuracy of the AR-KNEE system. The results indicated that the AR-KNEE system had accuracy comparable to other conventional navigation systems in terms of varus/valgus, posterior slope, and internal/external rotation angles (Stöckl et al. 2004; Pitto et al. 2006; Rosenberger et al. 2008). The mean absolute differences in previous studies ranged from 0.5° to 1.2° in varus/valgus angle and from 0.7° to 1.4° in posterior slope (Hasegawa et al. 2013; Tsukeoka et al. 2018; Feichtinger et al. 2018). Although the mean deference of 0.6 mm ± 0.7 mm in terms of the thickness of resected bone was also comparable to that of the conventional navigation system (Bäthis et al. 2005), we believe that the value of the mean difference can represent an unacceptable error for correct balancing during TKA. A recent cadaver study using robotically assisted technique showed more accurate results: the mean difference between planned bone resections and bone resections measured with calipers was 0.15 mm ± 1.08 mm at the lateral tibia plateau (Parratte et al. 2019). Therefore, at this time, we cannot recommend the AR-KNEE system as a means of reliable navigation for the thickness of the resected tibia.

### Limitations

This pilot study had several limitations. The materials used in this study were sawbones without soft tissue. The accuracy of registration touching bony landmarks through soft tissues was biased by the thickness of the soft tissue (Tsukada and Wakui 2010). This preclinical study cannot provide surgeons with information on whether the skin incision should be extended or not when using AR-KNEE in the clinical setting. Verification of the AR-KNEE system in patients is warranted.

All bone resections were performed by one surgeon in this study. The accuracy of bone resection may depend on the experience of the surgeon (Kazarian et al. 2019). Multi-surgeon studies will provide more robust external validity.

The AR-KNEE system may have a learning curve similar to other navigation systems for TKA (Jenny et al. 2008). It is possible that gaining experience with the AR-KNEE system would provide more accurate bone resection.

## Conclusions

Applying the AR-KNEE system to bone resection of the proximal tibia, the absolute values of differences between the values displayed on the smartphone screen and the measurement values obtained using CT were 0.5° ± 0.2°, 0.8° ± 0.9°, and 1.8° ± 1.5° in varus/valgus, posterior slope, and internal/external rotation angles, respectively. The AR-KNEE system may become a useful alternative navigation system for TKA.

## Data Availability

The datasets used and/or analyzed during the current study are not publicly available. Data are however available from the corresponding author on reasonable request.

## Abbreviations

AR: Augmented Reality
CT: Computed Tomography
DICOM: Digital Imaging and Communications in Medicine
THA: Total Hip Arthroplasty
TKA: Total Knee Arthroplasty
